# Mapping the Evidence for Measuring Energy Expenditure and Indicating Hypermetabolism in Motor Neuron Disease: A Scoping Review

**DOI:** 10.1093/nutrit/nuae118

**Published:** 2024-10-07

**Authors:** Sarah A Roscoe, Scott P Allen, Christopher J McDermott, Theocharis Stavroulakis

**Affiliations:** Division of Neuroscience, School of Medicine and Population Health, Sheffield Institute for Translational Neuroscience, The University of Sheffield, Sheffield S10 2HQ, United Kingdom; Division of Neuroscience, School of Medicine and Population Health, Sheffield Institute for Translational Neuroscience, The University of Sheffield, Sheffield S10 2HQ, United Kingdom; Division of Neuroscience, School of Medicine and Population Health, Sheffield Institute for Translational Neuroscience, The University of Sheffield, Sheffield S10 2HQ, United Kingdom; Division of Neuroscience, School of Medicine and Population Health, Sheffield Institute for Translational Neuroscience, The University of Sheffield, Sheffield S10 2HQ, United Kingdom

**Keywords:** motor neuron(e) disease, hypermetabolism, malnutrition, resting energy expenditure, total daily energy expenditure, indirect calorimetry, doubly labelled water, predictive energy equations

## Abstract

**Objective:**

To map the international methods used to measure energy expenditure of adults living with motor neuron disease (MND) and to highlight discrepancies when indicating hypermetabolism in the MND literature.

**Background:**

A decline in the nutritional status of patients is associated with exacerbated weight loss and shortened survival. Assessments of energy expenditure, using a variety of methods, are important to ensure an adequate energy intake to prevent malnutrition-associated weight loss. Assessments of energy expenditure are also commonly used to indicate hypermetabolism in MND, although these approaches may not be optimal.

**Methods:**

A protocol based on the Preferred Reporting Items for Systematic Reviews and Meta-analyses extension for Scoping Reviews Guidelines was developed. Three electronic databases (Medline [Ovid], CINAHL [EBSCO], and Web of Science) were exhaustively searched. Identified publications were systematically screened according to predefined PICOS eligibility criteria. The primary outcome was the identification of methods used to measure energy expenditure in MND. The secondary outcome was the identification of applications of energy expenditure assessments to indicate hypermetabolism in MND.

**Results:**

Thirty-two observational primary research publications were identified. Thirteen (40.6%) were longitudinal in design, with data on repeated measurements of energy expenditure presented in 3 (9.4%). Thirteen (40.6%) were case-control studies, of which 11 use a matched control group. Pulmonary function was used to assess eligibility in 10 publications. Energy expenditure was measured using indirect calorimetry (IC) in 31 studies. Discrepancies in the durations of fasted, measurement, and washout periods were observed. Of all included publications, 50% used assessments of resting energy expenditure to identify hypermetabolism. Bioelectrical impedance analysis was used to assess body composition alongside energy expenditure in 93.8% of publications.

**Conclusions:**

Resting energy expenditure is most frequently measured using an open-circuit IC system. However, there is a lack of a standardized, validated protocol for the conduct and reporting of IC and metabolic status in patients with MND.

## INTRODUCTION

Motor neuron disease (MND) encompasses a heterogeneous group of progressive neurodegenerative motor syndromes with a global prevalence of 3.37 per 100 000 people.^1^ MND is incurable, with death typically occurring from respiratory failure approximately 2–3 years after diagnosis.^2^^,^^3^ Amyotrophic lateral sclerosis (ALS) is the most common MND phenotype, comprising 65%–85% of MND cases.^4^ The terms MND and ALS are often used interchangeably in the international literature.

The term *nutritional status* can be defined as the condition of an individual’s health in relation to the intake and utilization of nutrients.^5^ A suboptimal caloric intake has been reported in 70%–94% of people living with MND, and this can lead to an energy imbalance and a decline in nutritional status.^6^^,^^7^ This is most commonly due to the presence of dysphagia and mastication weakness, with up to 30% of people living with MND reported to present with a reduced ability to swallow at diagnosis.^8^ Symptoms secondary to progressive, denervation-induced muscle weakness, such as a reduced mobility and/or dexterity, may cause difficulties with preparing and consuming food and/or drinks.^8^^,^^9^ This may be particularly challenging for patients without adequate home care and support. Other factors, such as a reduced appetite,^10^ fear of choking, as well as feelings of embarrassment about eating in public may also lead to food avoidance and anorexia.^11^ A decline in nutritional status can lead to irreversible protein-energy malnutrition.^12^ This is estimated to affect between 16% and 55% of people living with MND and is associated with a 3.5-fold increased risk of death.^6^^,^^13^

The accurate determination of an individual’s total daily energy expenditure (TDEE; an estimate of how many calories the human body burns over a 24-hour period [kcal/day]) is important to quantify nutritional energy requirements and provide informed energy intake goals for patients. In healthy adults, resting energy expenditure (REE; the minimum [nonactive] energy the human body needs to function at rest over 24 hours [kcal/day] including activities such as respiration, circulation, organ function, macronutrient utilization, and thermoregulation^14^) constitutes approximately 60%–70% of TDEE, with physical activity levels and dietary-induced thermogenesis composing the remaining 30%–40% of TDEE.^15^ The biggest determinant of REE is thought to be the proportion of fat-free mass (FFM) owing to the inclusion of metabolically active tissue,^16^^,^^17^ with other factors such as sex, age, and the regulation of energy homeostasis by the central nervous system also known to influence REE.^18^

### Assessment of Energy Expenditure

#### Total Daily Energy Expenditure

TDEE can either be measured directly or derived using independent assessments of REE, physical activity levels,^19^^,^^20^ and dietary-induced thermogenesis (TDEE = REE + physical activity levels + dietary induced thermogenesis).^21^^,^^22^ The doubly labelled water (DLW) method is considered to be the gold standard for directly measuring TDEE and total body water. Because fat mass (FM) is free of water, and the hydration of FFM remains constant (73%–80%) in healthy individuals,^23^^,^^24^ measurements of total body water using DLW can be used to estimate the proportion of FFM of an individual.^25^^,^^26^ The DLW method involves the oral or percutaneous administration of heavy hydrogen (^2^H) and oxygen (^18^O) isotopes followed by the subsequent analysis of carbon dioxide (CO_2_) as a urinary byproduct.^26^ However, the limited availability and high costs associated with the use of isotopes, as well as the complex and arduous process of urinary collection, processing, and analysis, mean this approach is less than ideal in a clinical setting.

#### Resting Energy Expenditure

REE can either be indirectly measured or predicted. Indirect calorimetry (IC) systems estimate respiratory gaseous exchange by measuring volumes of inspired oxygen (O_2_) and/or expired carbon dioxide (CO_2_) to derive measurements of REE (mREE) using the Weir equation.^27^^,^^28^ IC can be applied using different methods, such as through the use of mixing chambers (eg, Douglas bags),^29^ or open-circuit systems, which require a continuous air flow through a canopy hood or facemask measured over an aggregation interval.^30^ Regardless of the choice of method, limitations when using IC include the time and allocation of staffing to complete the testing, as well as the requirement of a mandatory overnight fast and rested period ahead of each measurement. This may be practically challenging in clinical studies and a possible burden on patients; however, it is important not to deviate from this requirement. It is also important to be aware of some assumptions inherent to how REE is calculated using IC. For example, it is assumed that the oxidation of fat, glucose, or protein can be calculated using a fixed ratio between O_2_ consumption and CO_2_ production.^30^^,^^31^

REE is most often predicted (pREE) in day-to-day clinical practice by equations developed from data on (mostly) healthy or patient groups.^32^ These equations most often incorporate combinations of age, weight, and height of an individual (eg, the Harris-Benedict [HB] equation).^33^ Predictive equations may also include assessments of FM and FFM independently estimated using technologies such as air displacement plethysmography (ADP) or bioelectrical impedance analysis (BIA) (eg, the Siri or Nelson equations).^34^^,^^35^ However, many predictive energy equations may not be suitable for use in patient cohorts that do not meet the inherent assumptions underlying the components of these predictive equations, such as in MND.^36^

### Hypermetabolism

There is growing interest in the stratification of individuals living with MND by metabolic status (ie, hypermetabolic, normometabolic, or hypometabolic). In the MND literature, hypermetabolism is defined as a higher-than-predicted REE for age, weight, and sex (calculated as the ratio of mREE to pREE).^17^^,^^37^ Approximately 50%–68% of people living with MND are estimated to be hypermetabolic,^17^^,^^37–40^ and evidence suggests that this state is associated with a faster rate of functional decline and shorter survival.^7^^,^^10^^,^^13^^,^^38^^,^^40^^,^^41^ Hypermetabolism in people living with MND is surprising, due to reductions in FFM often observed in the same individuals.^7^^,^^17^ It has been suggested that muscular fasciculations,^17^ increased respiratory demand,^7^ or defective mitochondria^42^ may also play a role, as reviewed by Dupuis et al^43^ and Perera et al.^44^

### Aim

Our aim for this scoping review was to map the international methods used to measure energy expenditure in adults living with MND, as well as to highlight the fundamental discrepancies when indicating hypermetabolism in the MND literature.

## METHODS

This scoping review was conducted following the 5-step framework outlined by Arksey and O’Malley^45^: (1) identification of the research question; (2) identification of primary research literature; (3) study selection; (4) data extraction; and (5) data synthesis.

### Identification of the Research Question

We sought to answer the following research question: What methods (ie, devices, protocols, equations, and outcome measures) have been used to measure energy expenditure (resting and total) in people living with MND? The objectives were defined according to the Population, Intervention, Comparator, Outcome, and Study Design (PICOS) framework (Table S1).^46^

### Identification of Primary Research Literature

We considered articles reporting on studies that measured energy expenditure in adults living with MND. This included articles on randomized controlled trials and analytical observational studies, prospective and retrospective cohort studies, case-control studies, cross-sectional studies and longitudinal studies. An exhaustive search of 3 major biomedical and health sciences databases (MEDLINE via Ovid, CINAHL via EBSCO, and Web of Science) was undertaken to identify primary research articles on the topic. The final database search was concluded on April 17, 2024. The search strategy, including all identified keywords and index terms, was developed in MEDLINE and subsequently adapted for CINAHL and Web of Science (Table S2). Keyword terms were optimized using wild cards and truncations and combined with Medical Subject Headings using Boolean operators. Only articles reporting on studies conducted with humans and published in the English language were included. Search results were not limited by publication date. Reference lists of key articles were screened by hand, and “cited by” articles on PubMed were used to identify additional articles.

All identified citations were collated and uploaded into Mendeley Reference Manager (version 2.107.0) and duplicates removed. One member of the research team systematically screened titles, abstracts and full text for eligibility according to the PICOS eligibility criteria (Table S1). To minimize bias, a second member of the research team also assessed all titles and abstracts. Discrepancies were resolved by discussion within the research team.

### Terminology and Definitions

Because of the variability of terminology used across the articles included in this review, estimations, calculations or predictions of REE will be referred to as pREE. Any terminology related to determining the accuracy or bias of predictions against measurements of REE are referred to as REE variation. All information relating to identifying the threshold (ie, cutoff point) of hypermetabolism (ie, change in REE, REE variation, metabolic index) is presented using the term metabolic index (MI). Presentation of the MI thresholds in this review is dependent on the specific equation applied to examine the ratio of mREE and pREE: for example, some may calculate this as [(mREE – pREE)/pREE] × 100 at a threshold of ≥10% or as (mREE – pREE) × 100 at a threshold of ≥110%.

## RESULTS

### Data Extraction

**Figure 1.**
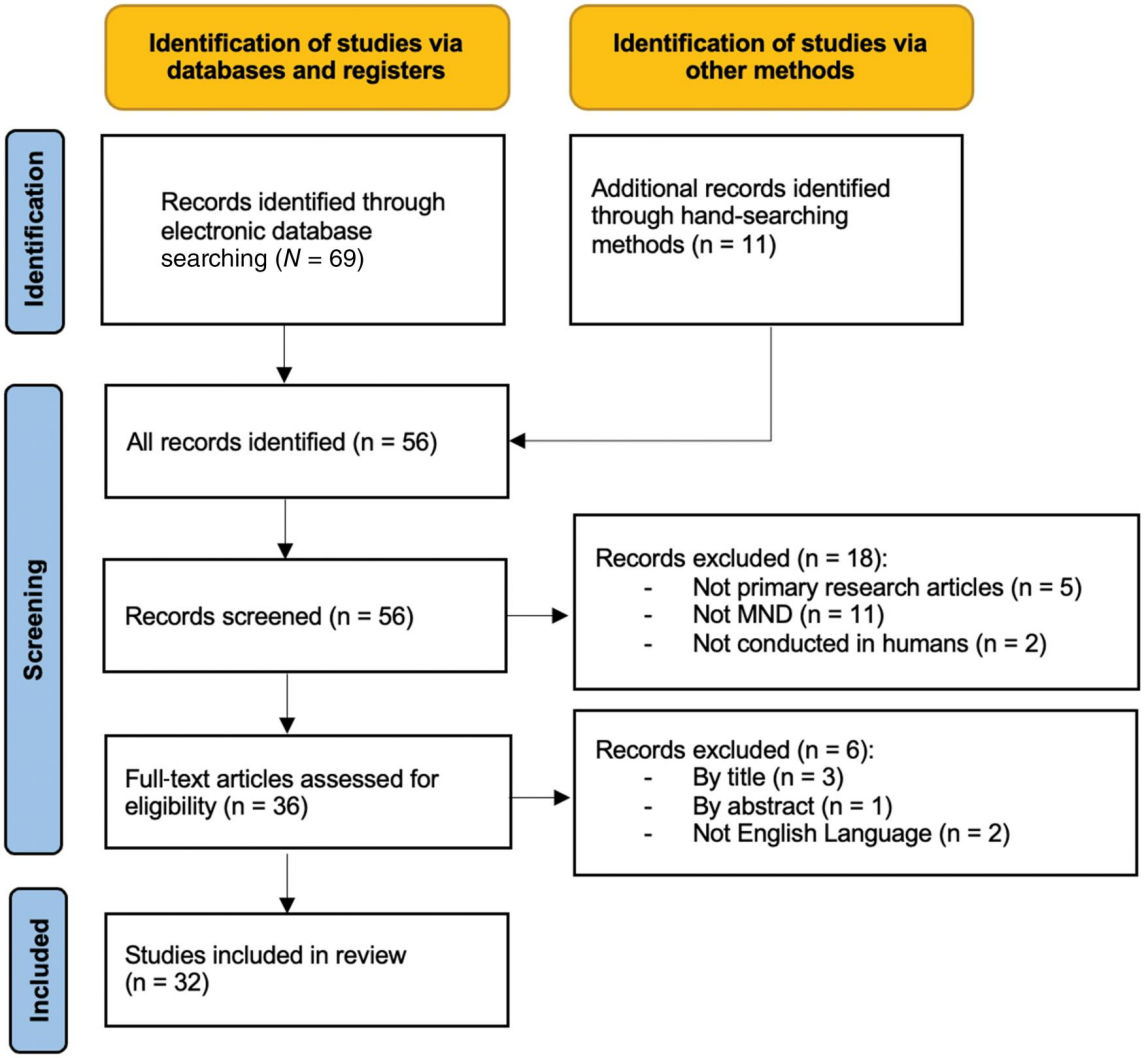
PRISMA Flow Diagram of the Study Selection Process. MND, motor neuron disease.

The search and study inclusion process is presented in a Preferred Reporting Items for Systematic Reviews and Meta-analyses extension for Scoping Review (PRISMA-ScR) flow diagram (Figure 1).

A total of 32 primary research articles were identified that met the acceptance criteria and were highly relevant to the research question (Table 1).^7^^,^^17^^,^^36–40^^,^^47–71^ Data were extracted using a data extraction tool developed by the authors, including study population demographics, study design, aims, and key findings relevant to the research question. In all instances, data were extracted only if explicitly stated within the text.

**Table 1.** Articles Included in the Scoping Review

Publication details/study identifiers			Study design				Cohort characteristics				Assessment method		Pulmonary function		
Identifier (reference no.)	First author, y	Country	No. of Study sites	Case-control or cohort	Prospective or retrospective	Cross-sectional or longitudinal	No. of participants	Age ( median), y	Sex (no. of F/M)	Control group (no.)	Energy expenditure measurement	Body composition assessment	Conducted?	Method	Inclusion/exclusion criteria?
1^47^	Nau et al (1995)	USA	Single	Case-control	Prospective	Longitudinal	12	MND 51.3 ± 12.7 Ctrl: 50.9 ± 12.3	MND: 0/12 Ctrl: 0/6	Yes+ (6)	IC	DEXA	No		
2^7^	Kasarskis et al (1996)	USA	Single	Cohort	Prospective	Longitudinal	16	58	8/8	No	IC	ANTH, BIA	Yes	FVC	No
3^17^	Desport et al (2001)	France	Single	Case-control	Prospective	Cross-sectional	62	MND: 63 ± 11 Ctrl: 66 ± 3	MND: 30/32 Ctrl: -	Yes (31)	IC	BIA	Yes	VC	
4^48^	Sherman et al (2004)	USA	Single	Cohort	Prospective	Cross-sectional	34	61.7 ± 8.85	18/16	No	IC	BIA	No		
5^49^	Desport et al (2005)	France	Single	Cohort	Prospective	Longitudinal	168	–	0.97 (5/163)	No	IC	BIA	Yes	FVC	
6^37^	Bouteloup et al (2009)	France	Multi	Cohort	Prospective	Longitudinal	61	64.3 ± 9.9	31/30	No	IC	DEXA	Yes	SVC, FVC, PEFR	
7^39^	Funalot et al (2009)	France	Single	Case-control	Prospective	Cross-sectional	11	fALS: 60.7 ± 8.8	fALS: 5/6	Yes+ (33)	IC	BIA	Yes	FVC	
								sALS: 60.4 ± 8.7	sALS: 15/18						
8^50^	Vaisman et al (2009)	Israel	Single	Case-control	Prospective	Longitudinal	33	MND: 59 ± 12.6	MND: 11/22	Yes+ (33)	IC	DEXA	No		
								Ctrl: 57.8 ± 12.3	Ctrl: 11/22						
9^51^	Siirala et al (2010)	Finland	Single	Cohort	Prospective	Longitudinal	5	55^a^	1/4	No	IC		Yes	TIPPV	Permanently on TIPPV
10^52^	Ellis et al (2011)	USA	Single	Cohort	Prospective	Cross-sectional	56	54.89 ± 11.98	25/31	No	IC	ANTH, BIA	Yes	FVC	
11^53^	Ichihara et al (2012)	Japan	Single	Cohort	Prospective	Cross-sectional	10	66 ± 11	3/7	No	DLW, Douglas bag	DLW	No		
12^54^	Georges et al (2014)	France	Single	Cohort	Prospective	Cross-sectional	16	68^a^	4/12	No	IC		Yes		Using NIV for 24 h to 3 mo
13^55^	Kasarskis et al (2014)	USA	Single	Cohort	Prospective	Longitudinal	80	58.7 ± 11.9	28/52	No	IC and DLW	BIA^b^	Yes	FVC	FVC ≥50% of predicted
14^56^	Shimizu et al (2017)	Japan	Single	Cohort	Prospective	Cross-sectional	26	64.5 (62.1–70.0)	13/13	No	DLW	DLW	Yes	FVC	Exclusion of Pt receiving ventilatory support
15^40^	Jésus et al (2018)	France	Single	Cohort	Prospective	Longitudinal	315	65.9 (56.5–73.7)	154/161	No	IC	ANTH, BIA	Yes	SVC, FVC, SNIF	
16^57^	Lunetta et al (2018)	Italy	Single	Case-control	Prospective	Cross-sectional	50	MND: 66 ± 9.81 Ctrl: 62 ± 12.15	MND: 16/34 Ctrl: 14/18	Yes+ (32)	IC	BIA	Yes	%FVC, blood gas analysis (pCO_2_/${\text{HCO}}_{2}^{–}$)	Exclusion of Pt receiving ventilatory support
17^38^	Steyn et al (2018)	Australia	Single	Case-control	Prospective	Longitudinal	58	MND: 61 ± 8 Ctrl: 59 ± 8	MND: 20/38 Ctrl: 21/37	Yes+ (58)	IC	ADP	Yes	FVC	FVC <60%
18^58^	Jésus et al (2019)	France	Single	Cohort	Prospective	Cross-sectional	315	65.9 (56.5–73.7)	154/161	No	IC	ANTH, BIA	Yes		
19^59^	Jésus et al (2020)	France	Single	Cohort	Prospective	Cross-sectional	315	66.6 (56.9–74.1)	154/161	No	IC	ANTH, BIA	Yes	FVC	
20^60^	Ngo et al (2020)	Australia	Single	Case-control	Prospective	Longitudinal	49	61.24 ± 8.81	15/34	Yes+ (51)	IC	ANTH, ADP	Yes	FVC	<60% FVC
21^61^	Steyn et al (2020)	Australia	Single	Case-control	Prospective	Cross-sectional	18	55.4 ± 7.2	4/14	Yes+ (11)	IC	ADP	Yes	FVC	
22^62^	Fayemendy et al (2021)	France	Multi	Case-control	Prospective	Cross-sectional	287	MND: 66.4 (56.7–73.1) Ctrl: 75.0 (68.5–86.0)	MND: 142/145 Ctrl: 35/40	Yes (75)	IC	ANTH, BIA	No		
23^63^	Kurihara et al (2021)	Japan	Single	Cohort	Retrospective	Cross-sectional	42	70 (61–74)	20/22	No	IC	BIA	Yes	FVC, FEV, tidal volume	
24^64^	Nakamura et al (2021)	Japan	Single	Cohort	Retrospective	Cross-sectional	48	71 (65–75)	23/25	No	IC	BIA	Yes	PEFR, VC	Exclusion of Pt receiving ventilatory support
25^65^	Cattaneo et al (2022)	Italy; France	Multi	Cohort	Retrospective	Longitudinal	847	63.79^a^	375/472	No	IC	BIA	Yes	FVC	NIV >16 h/d/invasive ventilation
26^66^	He et al (2022)	China	Single	Case-control	Prospective	Longitudinal	93	MND: 53.0 ± 10.1	MND: 32/61	Yes+ (147)	IC	BIA	Yes	FVC	
								Ctrl: 51.4 ± 11.6	Ctrl: 50/97						
27^67^	Nakamura et al (2022)	Japan	Single	Cohort	Retrospective	Cross-sectional	78	71 (66–75)	40/38	No	IC	BIA	Yes	VC	
28^68^	Dorst et al (2023)	Germany, Sweden	Multi	Case-control	Prospective	Longitudinal	60	MND: 48.7 ± 14.9	MND: 36/24	Yes+ (73)	IC	BIA	No		
								Ctrl: 47.2 ± 12.9	Ctrl: 39/34						
29^69^	Tandan et al (2023)	USA	Multi	Case-control	Prospective	Cross-sectional	10	MND: 55.9 ± 10.2	MND: 2/8	Yes+ (10)	IC, DLW	DEXA	Yes	FVC	Inability to lie supine
								Ctrl: 58.4 ± 6.8	Ctrl: 2/8						
30^36^	Roscoe et al (2023)	UK	Single	Cohort	Prospective	Cross-sectional	16	62 ± 12.1	0/16	No	IC	ANTH	No		
31^70^	Janse van Mantgem et al (2024)	The Netherlands	Single	Cohort	Prospective	Cross-sectional	140	62 ± 10.3	51/89	No	IC	ADP, BIA	Yes	FVC	Permanent assisted ventilation
32^71^	Holdom et al (2024)	Australia, the Netherlands, China	Multi	Case-control	Prospective	Cross-sectional	606						No		
		Australia					140	60.42 ± 9.93	39/101	Yes+ (154)	IC	ADP			
		The Netherlands					79	59.95 ± 10.11	26/53	Yes+ (37)	IC	ADP			
		China					67	51.95 ± 10.41	27/40	Yes (129)	IC	BIA			

Data presented as mean ± SD or median (IQR), as reported in the primary literature.

a Median was presented without IQR.

b It is worth noting that Kasarskis et al^55^ detail the use of bioelectrical spectroscopy; however, for purposes of this review, all bioelectric impedance analyses are grouped under BIA.

*Abbreviations:* ADP, air displacement plethysmography; ANTH, anthropometric measurement; BIA, bioimpedance analysis; Ctrl, control; DEXA, dual-energy X-ray absorptiometry; DLW, doubly labelled water; F, female; FEV, forced expiratory volume; FVC, forced vital capacity; IC, indirect calorimetry; M, male; MND, motor neuron disease; NIV, noninvasive ventilation; pCO_2_, partial pressure of carbon dioxide; PEFR, peak expiratory flow rate; Pt, participant; SNIF, sniff nasal-inspiratory force; SVC, slow vital capacity; TIPPV, tracheostomy and intermittent positive pressure ventilation; VC, vital capacity; Yes+, sex and age-matched control group; UK, United Kingdom; USA, United States of America; –, data not reported.

### Study Characteristics

The included articles were published over a 29-year span (1995–2024) across settings in 13 countries, with approximately one-third of the included literature published in France (*n* = 10 of 32; 31.3%).^17^^,^^37^^,^^39^^,^^40^^,^^49^^,^^54^^,^^58^^,^^59^^,^^62^^,^^65^ It should be noted that 3 of these articles^40^^,^^58^^,^^59^ were published from the same study; however, different data from this study were presented in each article. This review, therefore, refers to data extracted from individual articles, rather than studies. All studies reported in the included articles were observational in design. Thirteen articles (40.6%) were longitudinal; however, cross-sectional data relating to energy expenditure were reported in the majority of articles, with longitudinal energy expenditure data presented in 3 articles.^37^^,^^49^^,^^50^ Thirteen articles (40.6%) were about case-control studies,^17^^,^^38^^,^^39^^,^^47^^,^^50^^,^^57^^,^^60–62^^,^^66^^,^^68^^,^^69^^,^^71^ of which 11 used an age- and/or sex-matched control group.^38^^,^^39^^,^^47^^,^^50^^,^^57^^,^^60^^,^^61^^,^^66^^,^^68^^,^^69^^,^^71^ Matched control participants were healthy individuals except in one instance where the metabolic state of patients with sporadic ALS was compared with sporadic ALS cases.^39^ Individual study characteristics are outlined in Table 1. Twenty-four articles (75%) included an assessment of pulmonary function, of which 10 articles included an assessment of pulmonary function as an exclusion criterion (Table 1).

### Measurement of Energy Expenditure

Thirty-one articles (96.9%) measured energy expenditure using IC: 30 used open-circuit systems, and 1 used Douglas bags (Table 1).^53^  Table 2 details the reported characteristics of the open-circuit IC devices (type and style of calorimeter), protocol (fasted period, body position, duration of recording) and outcome measurements (mREE, volume of carbon dioxide expired [VCO_2_], volume of oxygen inspired [VO_2_], and respiratory quotient [RQ]). Data were extracted from citations in the included articles that referenced standardized protocols published elsewhere, if appropriate.

**Table 2.** Summary of Open-Circuit Indirect Calorimetry Protocol Data and Devices Reported in the Included Articles

Article identifier (reference no.)		Fasting duration (h)	Body position during measurement	Resting period (min)	Washout period (min)	Duration of recording (min)	CV (%)	VO_2_ (mL/min)	VCO_2_ (mL/min)	mREE (kcal/24 h)	RQ	Device, manufacturer	Mode
1^47^		–	–	–	–	≥20	–	–	–	–	–	Cybermedic, Metascope	–
2^7^		Overnight	–	–	–	–	Stable plateau	–	–	–	0.81 ± 0.03	Horizon, Beckman Instruments Inc	–
3^17^		≥10	Supine or semi-seated	≥20	–	20	“Stable plateau”	–	–	1561.6 ± 342.3	0.81 ± 0.04	Deltatrac II, Datex Engström	Canopy hood
4^48^		Overnight	Reclined	–	5	20	<5	–	–	Ventilated: 1654.9 ± 362.9	–	Cybermedic, Metascope	–
										Not ventilated: 1340.8 ± 471.6			
5^49^		≥10	Supine or semi-seated	≥20	–	20	Stable plateau	–	–	1521.9 ± 307.5	–	Deltatrac II, Datex Engström	Canopy hood
6^37^		Overnight	Supine or semi-seated	20	–	30–45	“Stable plateau”	–	–	1449.0 ± 300.7	–	Deltatrac II, Datex Engström	Canopy hood
7^39^		≥10	Supine or semi-seated	20–30	–	20	Stable plateau	–	–	fALS: 1784 ± 340	–	Deltatrac II, Datex Engström	Canopy hood
										sALS: 1582 ± 300			
8^50^		12	Supine	20	10	60	< 3	–	–	1467 ± 218	0.81 ± 0.06	Deltatrac II, Datex Engström	Canopy hood
9^51^		12	Supine	–	–	30	VO_2_: <10 RQ: <5	165 (± 25)	137 (± 24)	1130 ± 170 1060 (960–1480)	0.82 ± 0.08	Deltatrac II, Datex Engström	Canopy hood
10^52^		–	–	–	10	30	–	–	–	1488.84 ± 326.05	–	Vmax Spectra V29N, SensorMedics corporation	Canopy hood
12^54^		Overnight	Semi-seated	20	–	15	<5	–	–	Spontaneous breathing: 1197.3 (1054.7–1402.6)	–	Quark RMR, Cosmed	Oronasal mask
										NIV: 1149.2 (970.8–1309.5)			
13^55^		Overnight	–	–	–	–	–	–	–	1539 ± 366	–		
15^40^		12	Supine	–	–	–	–	–	–	1503 (1290–1698)	–	Quark RMR, Cosmed	Canopy hood
16^57^		–	–	–	–	–	–	–	–	1413.7 ± 314.9	–		
17^38^		12	35°	10	5	15	–	–	–	–	–	Quark RMR, Cosmed	Canopy hood
18^58^		12	Supine	–	–	–	–	–	–	1514 ± 298.7 1503 (1290–1698)	–	Quark RMR, Cosmed	Canopy hood
19^59^		12	Supine	–	–	30	–	–	–	1503 (1290–1698)	–	Quark RMR, Cosmed	Canopy hood
20^60^		12	35°	10	5	15	–	–	–	1604 ± 470	–	Quark RMR, Cosmed	Canopy hood
21^61^		12	35°	10	5	15	–	–	–	1809 ± 336.2	–	Quark RMR, Cosmed	Canopy hood
22^62^		12	Supine	–	–	–	–	–	–	1500 (1290–1693)	–	Deltatrac II, Datex Engström Quark RMR, Cosmed	Canopy hood –
23^63^		Overnight	Supine	30	–	10	–	–	–	1254 (1082–1500)	0.84 (0.81–0.91)	Aeromonitor AE310S, Minato Medical Science	Oronasal mask
24^64^		Overnight	Supine	30	–	10	–	–	–	–	–	Aeromonitor AE310S, Minato Medical Science	Oronasal mask
25^65^		12	35°	10–20	5	20	Stable plateau	–	–	1430.00 (1239–1650)	–	Vmax Spectra V29N, SensorMedics corporation	Canopy hood
												Vyntus CPX, Carefusion	Canopy hood
26^66^		≥ 6	Semi-supine	–	5	16	Steady–state values (showing the least variability)	–	–	–	–	ULTIMACardio2, Medgraphics Corp	Oronasal mask
27^67^		–	–	–	–	–	–	–	–	–	–	Aeromonitor AE310S, Minato Medical Science	Oronasal mask
28^68^		≥5	Supine	20	5	16	<10	–	–	1598 (1376–1885)		Quark RMR, Cosmed	–
29^69^		Overnight	–	–	–	–	–	–	–	1881 ± 253	–	Deltatrac II, Datex Engström	Canopy hood
30^36^		3.5	30°	60	5	20	≤5	234.05 ± 37.56	211.87 ± 31.36	1642 ± 258	–	GEMNutrition	Canopy hood
31^70^		10	–	–	–	20	–	–	–	–	–	Quark RMR, Cosmed	Canopy hood
32^71^	Australia	≥12	30–45°	–	5	20	–	–	–	1656 ± 410	–	Quark RMR/Q-NRG, Cosmed	Canopy hood
	Netherlands	≥12	30–45°		5	20				1747 ± 264		Quark RMR/Q-NRG, Cosmed	Canopy hood
	China	≥6	Semi-supine		≥5	≥16				1654 ± 418		ULTIMACardio2, Medgraphics Corp	

Data is presented as mean ± SD or median (IQR).

*Abbreviations:* CV, coefficient of variation; fALS, familial amyotrophic lateral sclerosis mREE, measured resting energy expenditure; NIV, noninvasive ventilation; VO_2_, volume of oxygen consumed; VCO_2_, volume of carbon dioxide expired; RQ, respiratory quotient; sALS, sporadic amyotrophic lateral sclerosis; –, data not reported.

Nine different devices were referenced across the 30 publications reporting on studies in which an open-circuit system was used (Table 2). Of note, 3 multicenter studies used different devices at each site.^62^^,^^65^^,^^71^ Where reported (*n* = 25), the majority of articles (*n* = 20; 80%) used a ventilated canopy hood setup, as opposed to an oronasal mask. Of the articles that reported fasting ahead of IC measurements (*n* = 26 of 30; 86.7%), the reported fasted periods ranged between 3.5 and 12 hours. The 8 articles (26.7%) that stated the occurrence of an overnight fast could not be quantified in terms of their duration in hours).^7^^,^^37^^,^^48^^,^^54^^,^^55^^,^^63^^,^^64^^,^^69^ Fourteen articles reported a rested period ahead of the calorimetry measurements,^17^^,^^36–39^^,^^49^^,^^50^^,^^54^^,^^60^^,^^61^^,^^63–65^^,^^68^ which ranged between 10 and 60 minutes (Table 2). The reported duration of calorimetry assessment varied between 10 minutes and 1 hour, with washout periods (where data were discounted) reported in 11 articles, ranging between 5 and 10 minutes.^36^^,^^38^^,^^48^^,^^50^^,^^52^^,^^60^^,^^61^^,^^65^^,^^66^^,^^68^^,^^71^ To demonstrate that data were collected over a steady state, the coefficient of variation (CV) value, reported in 6 articles, ranged from <3% to 10%;^36^^,^^48^^,^^50^^,^^51^^,^^54^^,^^68^ and 7 articles stated that a stable plateau or steady state was reached but did not state the CV.^7^^,^^17^^,^^37^^,^^39^^,^^49^^,^^65^^,^^66^ Of the 22 articles that provided information on body position, 6 provided the angle of the participant’s body during the measurement; this ranged between 30° and 45°.^36^^,^^38^^,^^60^^,^^61^^,^^65^^,^^71^ At least 1 outcome measurement (ie, VO_2_, VCO_2_, mREE, or respiratory quotient) was reported in 23 of the 30 articles (76.7%). However, there was no consistency when reporting the measures of central tendency (eg, the mean [SD], or median [IQR]) of these data.

Table 3 presents characteristics related to the conduct of DLW as reported in 4 articles across 2 research groups.^53^^,^^56^^; ^^55^^,^^69^ All studies included a urinary collection prior to the administration of DLW to a patient. Subsequent urinary collections ranged between 10 and 15 days, varying in frequency. The average measured TDEE using DLW ranged between 934 (SD ± 201) and 2844 (SD ± 319) kcal/day. The ratio of measured TDEE to mREE using IC was calculated in 2 articles.^53^^,^^55^

**Table 3.** Summary of Doubly Labelled Water Protocol Information Reported in the Included Articles

Article identifier	Oral dose	Measurement duration (d)	Frequency of urinary collections	Timing of urine collections	TDEE (kcal/d)	mREE (kcal/d)	TDEE/REE
11^53^	Per kg body weight: 0.14 g ^18^O 0.06 g ^2^H	14	6	Days 0 and 1, plus 4 samples at unspecified timing between days 2 and 14	934 ± 201	807 ± 116	1.14 ± 0.09
13^55^	Per kg body water: 0.120 g ^18^O 0.236 g ^2^H	10	4	Days 0, 1 (×2), 10 (×2)	2364 ± 647	1539 ± 366	1.5 ± 0.04
14^56^	Per kg body weight: 0.14 g ^18^O 0.06 g ^2^H	15	9	Days 0, 1, 2, 3, 8, 9, 13, 14, 15	1628 (1352–1865)	–	–
29^69^	Per kg body water: 0.120 g ^18^O 0.236 g ^2^H	10	3	Days 0, 1, 10	2844 ± 319	1881 ± 253	–

Continuous data are presented as mean ± SD or median (IQR).

*Abbreviations:* mREE, measured resting energy expenditure; REE, resting energy expenditure; TDEE, Total daily energy expenditure; ^2^H, heavy hydrogen; ^18^O, oxygen isotope; –, data not reported.

Table 4 presents the equations, thresholds, predictive energy equations, and results for all articles that assessed the REE variation and/or the percentage of accuracy within the study population (*n* = 14 of 32 articles [43.8%]).^17^^,^^36^^,^^37^^,^^48–52^^,^^55^^,^^57^^,^^58^^,^^62^^,^^63^^,^^71^ The HB equation^33^ was the most frequently used equation; it was referenced in all 14 publications. When assessed at a threshold of ±10%, pREE was reported to be accurate in 27.3%–70% of 5 study populations, regardless of the equation used.^36^^,^^50^^,^^52^^,^^58^^,^^71^

**Table 4.** Comparing pREE and mREE to Calculate the REE Variation and Accuracy (%)

Article identifier	mREE (kcal/24 h)	Equation	Acceptable threshold (%)	Predictive energy equation	pREE (kcal/24 h)	REE variation/bias (%)	Accurate (% of study population)
3^17^	1561.6 ± 342.3	–	–	HB^32^	1334 ± 234.7	–	–
4^48^	Ventilated: 1654.9 ± 362.9 Not ventilated: 1340.8 ± 471.6	(pREE – mREE)/mREE × 100	<20	HB^32^	Ventilated: 1461 Not ventilated: 1505	Average: 18.6 ± 14.9	67.6
				Fusco^71^	–	25.6 ± 23.8	–
				Ireton-Jones^72^	–	21.09 ± 17.5	–
				Weight-based	–	20.6 ± 14.3	–
5^49^	1521.9 ± 307.5	–	–	HB^32^	1334 ± 234.7	–	–
6^37^	1449 ± 300.7	–	–	HB^32^	1315.5 ± 242.2	–	–
8^50^	1467 ± 218	–	±10	HB^32^	–	–	51.5
9^51^	1060 (960–1480)	–	–	HB^32^	1580 (1190–2020)	–	–
				MSJ^73^	1557 (1399–1909)	–	–
				FAO/WHO/UNU^74^	1656 (1374–2039)	–	–
				Owen^75^	1726 (1183–1879)	–	–
				Fleisch^76^	1630 (1210–1938	–	–
10^52^	1488.84 ± 326.05	–	±10	HB^32^	1522 ± 39	3.7	52
				MSJ^73^	1431 ± 37	–2.7	63
				Ireton-Jones^72^	1660 ± 40	13.9	46
13^55^	1539 ± 366	–	–	HB^32^	1596 ± 283	–	–
				MSJ^73^	1523 ± 283	–	–
				Owen^75^	1589 ± 250	–	–
				Wang^77^	1315 ± 264	–	–
				Rosenbaum^78^	1508 ± 203	–	–
16^57^	1413.7 ± 314.9	–	–	HB^32^	1320.8 ± 202.1	–	–
18^58^	1514 ± 298.7	(pREE– mREE)/mREE × 100	± 10	HB^32^	1356 ± 222.2	–9.4	45.1
				HB^79^	1375 ± 212.8	–7.9	49.8
				World Schofield^80^	1381 ± 207.1	–7.1	43.5
				De Lorenzo^80^	1376 ± 224.9	–8.1	50.2
				Johnstone^84^	1326 ± 215.5	–11.1	36.9
				MSJ^73^	1285 ± 241.6	–14.8	27.3
				WHO/FAO/UNU^74^	1421 ± 213.2	–4.9	54.9
				Owen^75^	1418 ± 206.9	–4.3	57.5
				Fleisch^76^	1398 ± 189	–6.7	54.0
				Wang^77^	1281 ± 224	–14.3	32.1
				Rosenbaum^78^	1369 ± 178	–7.4	46.7
22^62^	1500 (1290–1693)	–	–	HB^32^	1327 (1195–1496)	–	–
23^63^	1254 (1082–1500)	–	–	HB^32^	1146 (1060–1275)	–	–
				Shimizu^55^	1660 (1531–1923)	–	–
30^36^	1642 ± 258	((pREE – mREE)/mREE) × 100	± 10	HB^32^	1655 ± 265	2.81 ± 20.81	31.3
				Henry^81^	1683 ± 231	4.51 ± 18.98	31.3
				kcal/kg/d^82^	1798 ± 249	8.00	58.3
32^71^	–	–	±10	HB^32^	–	Australia: 6.7	Australia: 62
						China: 46.6	China: 31
						The Netherlands: 85.1	The Netherlands: 70
				Sabounchi Structure 4^83^	–	Australia: 8.3	Australia: 67
						China: 43.0	China: 31
						The Netherlands: 126.2	The Netherlands: 65

Data presented as mean ± SD or median (IQR).

*Abbreviations:* FAO/WHO/UNU, Food and Agriculture Organization/World Health Organization/United Nations University; HB, Harris-Benedict; mREE, measured resting energy expenditure; MSJ, Mifflin-St Jeor; pREE, predicted resting energy expenditure; REE, resting energy expenditure.

### Determining Metabolic Status

#### Determining the Metabolic Index

In 20 articles, the MI was calculated by comparing pREE and mREE values (Table 5).^17^^,^^36–40^^,^^49^^,^^57^^,^^59–68^^,^^70^^,^^71^ Participants were classified as hypermetabolic or not depending on the selected metabolic index threshold chosen by the authors. Hypermetabolism was indicated in 6.4%–100% of the study populations included in these 20 articles, with prevalence varying depending on the predictive equation used and the chosen MI threshold. The majority of these articles (*n* = 14; 70%) compared mREE to pREE derived by the HB equation.^33^ Use of the HB^33^ equation at a metabolic index threshold of >10/110% indicated the prevalence of hypermetabolism varied between 37.5% and 100% across 9 articles (45%).^36^^,^^37^^,^^39^^,^^40^^,^^49^^,^^57^^,^^59^^,^^62^^,^^65^ When the MI threshold was increased to 20/120%, still using the HB equation,^33^ the prevalence of hypermetabolism ranged between 23.1% and 45.2% in 2 articles.^59^^,^^66^ Comparisons could not be drawn across articles in which the MI threshold was not stated.

**Table 5.** Calculation and Prevalence of Hypermetabolism, Using Predictive Energy Equations and the Metabolic Index Threshold

Article identifier	Predictive equation	Equation	Threshold (%)	Metabolic index (%)			Hypermetabolic participants (%)	
3^17^	HB^32^						67.7	
5^49^	HB^32^		110	14.1 ± 12.5			62.3	
6^37^	HB^32^	(mREE – pREE)/pREE	≥10	10.5 ± 10.9			47.54	
7^39^	HB^32^	mREE/pREE	110	fALS: 127 ± 9			fALS: 100	
				sALS: 112 ± 12			sALS: 52	
15^40^	HB^32^	[(mREE – pREE)/pREE] × 100	>10	11.8 (3.7 – 19.8)			55.24	
16^57^	HB^32^	(mREE – pREE)/pREE	≥ 10				52	
17^38^	Nelson^34^		120	115 ± 21			41	
19^59^							**10%**	**20%**
	HB^32^	(mREE – pREE)/pREE	10/20				55.2	23.1
	HB^79^						49.8	20.0
	World Schofield^80^						46.7	19.7
	De Lorenzo^80^						49.2	20.0
	Johnstone^84^						64.1	28.9
	MSJ^73^						72.7	47.9
	WHO/FAO^74^						38.4	14.9
	Owen^75^						35.2	14.6
	Fleisch^76^						44.4	16.2
	Wang^77^						67.6	42.9
	Rosenbaum^78^						49.1	22.6
	Nelson^34^						76.3	53.3
20^60^		mREE/pREE × 100		114.2 ± 22.51			45.5	
21^61^		mREE/pREE × 100	≥120	119.5 ± 9.6			38.9	
22^62^	HB^32^	[(mREE – pREE)/pREE] × 100	>10	11.5 (3.6–19.3)			55	
23^63^	HB^32^	mREE/pREE		1.07 (0.99–1.16)				
24^64^	LSTM	mREE/LSTM	≥38 kcal/kg	36.4 (34.4–40.5)			23.91	
25^65^	HB^32^	[(mREE – pREE)/pREE] × 100	≥10	7.0 (–2.0 to –15.94)			40	
26^66^	HB^32^	mREE/pREE	≥120	121.7 ± 38.0			45.2	
27^67^	LSTM	mREE/LSTM	≥38 kcal/kg	37.1 (34.5–41.2)			47	
28^68^	HB^32^	mREE/pREE		1.04 (0.98–1.13)				
30^36^	HB^32^	(mREE/pREE) × 100	≥110	101.04 ± 20.33 100.06 (80.90–113.32)			37.5	
	Henry^81^			98.62 ± 17.40 98.93 (81.77–112.65)			31.3	
	kcal/kg/d^82^			95.64			8.33	
31^70^	Sabounchi Structure 4^83^	(mREE/pREE) × 100	≥110/120	ADP: 108.2 ± 9.7			**110** ADP: 44.2	**120** ADP: 7.9
				BIA: 105.7 ± 10.4			BIA: 31.4	BIA: 6.4
				**Australia**	**China**	**The Netherlands**		
32^71^	HB^32^	mREE/pREE	>1 SD above mean value	1.02 ± 0.16	1.13 ± 0.23	1.09 ± 0.10		
	Sabounchi Structure 4^83^	mREE/pREE		1.04 ± 0.18	1.15 ± 0.22	1.10 ± 0.09		

Continuous data are presented as mean ± SD and/or median (IQR).

Abbreviations: HB, Harris-Benedict; fALS, familial amyotrophic lateral sclerosis; FAO, Food and Agriculture Organization; LSTM, lean soft-tissue mass; mREE, measured resting energy expenditure; MSJ, Mifflin-St Jeor; pREE, predicted resting energy expenditure; UNU, United Nations University; WHO, World Health Organization.

#### Considering Body Composition to Determine Metabolic Status

The body composition (ie, FM and FFM) of participants was assessed in 30 of 32 articles (93.8%) (Table 1). BIA was the most commonly reported approach for the assessment of body composition, used in 20 of the 30 articles (66.7%). Other methods of body composition assessment included anthropometric measurements (eg, triceps skinfold thickness, mid-upper arm circumference, arm muscle area) (*n* = 8 of 30);^7^^,^^36^^,^^40^^,^^52^^,^^58^^,^^59^^,^^60^^,^^62^ dual energy x-ray absorptiometry (*n* = 4);^37^^,^^47^^,^^50^^,^^69^ ADP (n = 5);^38^^,^^60^^,^^61^^,^^70^^,^^71^ and DLW (n = 2).^53^^,^^56^

Steyn et al^38^ assessed body composition using ADP to determine the effect of FM and FFM on the metabolic status of people living with MND. The acquired FM and FFM values in this study were subsequently entered into the Nelson predictive energy equation^35^ to predict REE. As a result, 41% of this cohort (*n* = 24 of 58) was classified as hypermetabolic (metabolic index: 115% [SD ± 21] at a threshold of 120%) (Table 5). This is lower than the proportion of study participants identified as hypermetabolic by Jésus et al^59^ (*n* = 168 of 315; 53.3%) when the same equation and metabolic index threshold were applied (Table 5).

Rather than incorporating assessments of body composition into predictive energy equations, Nakamura et al^64^^,^^67^ identified hypermetabolic participants by comparing mREE and lean soft tissue mass estimated by BIA. This identified 23.9%–47% of participants in their articles to be hypermetabolic (Table 5). Janse van Mantgem et al^70^ assessed FM and FFM in 140 patients with ALS, using both BIA and ADP. pREE was estimated by applying the Sabounchi Structure 4 formula.^84^ pREE was lower when using ADP-derived FM and FFM values (1577.9 kcal/day) compared with BIA-derived FM and FFM values (1619.9 kcal/day). As a result, a significant difference in the MI was observed (*P *=* *.048). In addition, the proportion of participants classified as hypermetabolic was increased when pREE was calculated using ADP, regardless of the metabolic index threshold (≥110% = ADP: 44.2%, BIA: 31.4%; ≥ 120% = ADP: 7.9%, BIA: 6.4%) (Table 5).^70^

## DISCUSSION

This review identified reported approaches to assess TDEE and REE in people living with MND. Four articles assessed the TDEE, using the DLW method, of a cohort of people living with MND.^53^^,^^55^^,^^56^^,^^69^ The DLW method provides a measure of the average total energy expended over 3–21 days, which provides a better estimate of habitual free-living energy expenditure. This may be more accurate than deriving TDEE from individual assessments of REE, physical activity, and thermogenic influences from the diet. However, clinical and research applications of DLW are often impractical due to the length of the observational period, requirement of multiple urinary sample collections, and the downstream, time-consuming isotope analysis.^26^

Kasarskis et al^55^ developed a new approach to estimate TDEE using MND-specific predictive energy equations. A physical activity factor of 1.5–1.6 was calculated by dividing measured TDEE (using DLW) by mREE (using IC). Statistical modelling using clinically accessible parameters led to the development of the “Model-6” equation, which incorporates the HB^33^ pREE equation and participant self-determined estimates of physical activity based on responses to 6 questions from the revised ALS functional rating scale (ALSFSRS-R), ALSFRS-6. The ALSFRS-6 score is calculated from the sum of questions: 1 (speech), 4 (handwriting), 6 (dress and self-care activities), 7 (turn in bed and adjust bed clothes), 8 (ability to walk) and 10 (shortness of breath) from the ALSFRS-R^86^) to assess physical function.^87^ However, Bland-Altman analysis in this study indicated a greater overestimation of predicted TDEE when measurements of TDEE using DLW were lower, and vice versa.^55^ The authors suggested this inaccuracy and variation were associated with inaccurate assessments of metabolic cost from physical activity using the ALSFRS-6 subscore, which requires further investigation.^86^

This review identified that IC using open-circuit systems is the most commonly used approach to assess REE in the current MND literature. Notwithstanding, there is a distinct lack of consistency in the reporting of IC protocols and related outcome measures in articles about people living with MND (Table 2). Although generic recommendations exist for the conduction of IC in healthy populations,^88^^,^^89^ these may not be applicable to MND cohorts, and robust evidence is lacking. In reality, it may be practically challenging to meet the generalized recommendations when conducting IC in patients with MND. For example, achieving a steady state (CV ≤10%) may not be possible because of disease-associated muscle rigidity, although this has not been reported in the MND literature.^90^ In addition, although it is important to facilitate a rested period ahead of IC measurement, an individual with a more severe disability will use more energy than an individual without mobility restrictions will when moving or transferring, increasing the mREE. Finally, the recommendation of a 5-hour fasted period as a minimum may be contentious, with evidence to suggest that the thermogenic influence wanes by 2–3 hours after eating.^91^^,^^92^ Although a shorter fasted period would be beneficial for IC studies by reducing participant burden and increasing the practicality of conducting IC, the evidence for this is not specific to MND, and further investigation is required to reduce additional variations and bias before modification in future study designs.

The lack of consistency when reporting measures of central tendency reduces the ability to compare measurements of REE across different cohorts of patients with MND. For example, of the 23 articles that reported values of mREE following IC, 14 presented the mean and SD, 7 presented the median and IQR, and 2 presented both (Table 2). Moreover, differences in the reporting of IC outcome measurements enables differential calculations and interpretations of mREE. For example, because mREE is derived from measurements of VO_2_ and VCO_2_ (mL/minute) using the Weir equation,^28^ VO_2_ is considered the more accurate outcome measurement from IC and should be presented alongside mREE. VO_2_ was reported alongside mREE in 2 of 32 articles (6.3%). Standardization of the reporting of these measurements would allow comparisons of mREE between articles, increasing transparency and allowing flexible analysis of multicohort articles. Moreover, reporting of participant characteristics, including sex, weight, height, and body composition (where assessed), would enable flexibility when retrospectively calculating the MI with different predictive energy equations across study populations. This is particularly pertinent when comparing international study populations in which demographics and body compositions influence the accuracy of pREE, as presented and discussed by Holdom et al.^71^ This should be a priority for all researchers investigating metabolic state in MND. The provision of data sharing would potentially enable the creation of a comprehensive, international database that could be used to perform meta-analysis and critically examine changes in mREE with disease stratification, for example.

### Drivers of Hypermetabolism

Because MND is a heterogeneous condition, the observed variability in the mREE may be attributed to the age, sex, FFM, disease stage, phenotype, or severity of the different study cohorts. For example, Funalot et al^39^ compared the metabolic parameters of individuals with familial ALS against those with sporadic ALS and found that mREE was lower in the sporadic cohort than those in the familial cohort (sporadic ALS: 1582, SD ± 300 kcal/day; familial ALS: 1881, SD ± 253 kcal/day). These results did not correlate with neurological or respiratory function and were irrespective of disease duration or severity. The authors proposed that this was associated with a defective energy homeostasis arising from mitochondrial uncoupling in muscular tissue.^39^

Further challenges with IC are associated with respiratory complications such as a weakening of the diaphragmatic and intercostal muscles, which is exacerbated in a supine position.^93^ Twenty-four articles (75%) in this review accounted for pulmonary function. Of these, 10 reported on studies that excluded participants with reduced respiratory function by either FVC score or ALSFRS-R respiratory subdomains. One study excluded participants unable to lie in a supine position for 1 hour.^69^ The “respiratory hypothesis” originates from a study conducted with 11 patients receiving mechanical ventilatory support and living with ALS who presented with weight gain and hypometabolism.^94^ It was hypothesized that energy requirements were decreased after alleviation of respiratory demands. This study did not meet the inclusion criteria (it was not published in English) defined for this scoping review (Table S1). Kasarskis et al^7^ suggested that an increasing metabolic index observed toward end of life was a result of increased energy demand from respiratory muscles, which may be decreased in those receiving noninvasive ventilation (NIV). This hypothesis was debated further when Sherman et al^48^ and Georges et al^54^ compared the mREE of patients with MND who were receiving NIV (mREE_NIV_) with those who were breathing spontaneously (mREE_BS_). Although Sherman et al^48^ reported that patients who were breathing spontaneously had a lower mREE than those with NIV (mREE_BS_: 1341, SD ± 472 kcal/day; mREE_NIV_ 1655, SD ± 363 kcal/day), Georges et al^54^ presented a significant reduction in the mREE of patients receiving mechanical ventilatory support compared with those breathing spontaneously. These contrasting results could be attributed to the difference of the mean BMI in the 2 cohorts (24.5 kg/m^2^^48^ vs 22 kg/m^2^^54^, respectively). Sherman et al^48^ also proposed that the counterintuitive increase in mREE_NIV_ could be related to an increased dietary thermogenesis resulting from recent refeeding as a result of gastrostomy insertion.

Consideration and adjustments should be applied when conducting IC for individuals requiring continuous ventilatory support or tracheotomy positive pressure ventilation.^51^^,^^53^^,^^54^ For example, although there is no evidence, to our knowledge, as to whether the participant’s body position during IC (ie, the angle of the head and torso) influences the measurements, it is important to consider that individuals with a decreased respiratory capacity may not be able to lay in a reclined or supine position, and this could potentially influence IC outcome measurements.

In a prospective, longitudinal, case-control study of 93 people living with MND and 147 matched healthy control participants, He et al^66^ proposed the concept of “dynamic alteration” of energy expenditure in MND. These researchers observed a continuous increase of the MI in the preclinical stage, a decline in the period after diagnosis, and a significant reduction between stages 1 and 5 of the King’s College Staging System (a 5-stage system based on the weakness or wasting of neurological regions^95^).^66^ Dorst et al^68^ supported this concept with their own findings from a prospective longitudinal study which compared the metabolic rate of 60 presymptomatic ALS gene carriers with that of 73 individuals from the same families without pathogenic mutations (Table 1). When REE was measured using IC (Table 2) and compared with pREE by applying the HB^33^ equation (Table 5), the presymptomatic ALS gene carriers had a lower mREE and MI, which increased with proximity to the expected disease onset.^68^

### Identification of Hypermetabolism

There is no consensus on the comparator, equation, threshold, or terminology by which to identify hypermetabolism in MND. This may explain not only the variation in the prevalence of hypermetabolism observed across the MND cohorts in the studies reported by the included articles but also the disparity in the prevalence of hypermetabolism observed between the MND and control cohorts. For example, when hypermetabolism was assessed by comparisons of mREE and predictive energy equations, the MI was significantly increased.^38^^,^^60^^,^^61^^,^^66^^,^^71^

This review has identified that the HB^33^ pREE equation is the most commonly used comparator against mREE when calculating the MI in cohorts of individuals living with MND (Table 5). We have previously criticized the suitability of applying the HB^33^ equation to indicate the state of hypermetabolism in an MND cohort.^36^ We observed that extreme body weight variations influence the prediction accuracy of REE (ie, the lighter the body weight of an individual, the greater the underestimation of pREE, and vice versa). An underprediction of pREE consequently leads to the calculation of a greater metabolic index, introducing a bias in the way patients may be classified as hypermetabolic.^36^ This influence may be exaggerated when compared with healthy cohorts, whose body composition may be more reflective of the cohort from which the predictive equations were derived.

Ellis et al^52^ suggested that predictive energy equations in general, not just the HB^33^ equation, may be more accurate in individuals with a “healthy” nutritional status, defined as a BMI of between 18 and 30 kg/m^2^. This may explain the discrepancy in the accuracy of each predictive equation presented in this review across different study cohorts, demonstrated by the range of REE variations (–14.8% to 13.9%) (Table 4). For example, although Ellis et al^52^ observed that the Mifflin-St Jeor equation was the most accurate equation in their study, with an average REE variation of –2.7% (accurate in 63% of the study population with an average BMI of 24.14 kg/m^2^), Jésus et al^58^ observed that the same equation had an average REE variation of –14.8%, accurate in only 27.3% of their study population with a median BMI of 24.2 kg/m^2^.

FFM is regarded as a contributing factor to REE.^96^ Therefore, because the proportions of FM and FFM for an individual living with MND often deviate from the expected ratios for sex, age, weight, and height, a plausible explanation for this inaccuracy is that MND cohorts do not follow the inherent assumptions underpinning the inclusion of weight in the predictive energy equations. Determining hypermetabolism using predictive equations that include estimates of body composition may be more suitable, therefore, for people living with MND. Holdom et al^71^ reported that FFM consistently contributes to mREE regardless of geographic location; therefore, predictive equations should consider FFM accounting for sex and age, where possible.

Proportions of FM and FFM were assessed using BIA in approximately two-thirds of articles included in this review. When the REE to FFM ratio of MND cohorts was compared to matched healthy control groups, the MI was significantly higher in the MND cohorts.^50^^,^^69^ Jésus et al^58^ developed an ALS-specific predictive equation for REE incorporating FFM and FM using BIA.^58^ It was suggested that this equation accurately estimated REE in 65% of the study population (at a threshold of ±10%); however, it would be interesting to know the proportion of this study population who were identified as hypermetabolic using this formula. This equation was not included in any other study in this review; therefore, further comparisons are not possible at this stage.

REE was underpredicted by the greatest margin when assessments of FM and FFM using BIA were entered into the Nelson equation by Jésus et al^59^ (data presented graphically in the article of Jésus et al). This also had the greatest influence on the metabolic index, with 76.3% of study population indicated to be hypermetabolic at a threshold of ≥10% (Table 5).^59^ Nakamura et al^64^^,^^67^ also used BIA to estimate FFM; however, FFM was not incorporated into a predictive equation. Rather, hypermetabolism was indicated by a ratio of ≥38 kcal/kg when mREE was compared with measurements of lean soft tissue mass (Table 5). This indicated hypermetabolism in 23.9%–47% of these study cohorts.^64^^,^^67^

It is important to factor in the stage of disease progression and severity of the study cohort when considering body composition, and to keep in mind that BIA is an indirect assessment of body composition that relies on derivation equations largely developed in healthy populations to calculate FM and FFM.^97^ Janse van Mantgem et al^70^ observed that predictions of REE, using BIA to assess FM and FFM, were lower than predictions of REE estimated using ADP. Steyn et al^38^ used FFM values, derived from ADP measurements, to predict REE; however, the accuracy of pREE was not reported and comparisons cannot be drawn between the findings of the 2 articles.

The statistical impact of using different thresholds and predictive equations to identify hypermetabolism is best exemplified in Table 3 of the 2020 Jésus et al article.^59^ That table demonstrates significant differences in the number of participants indicated to be hypermetabolic vs the metabolic index calculated using the HB^33^ equation at a threshold of 10%.^59^ Inappropriate use of predictive equations and thresholds can lead to the misclassification of hypermetabolism in people living with MND, which, in turn, can lead to implications such as exclusion from clinical research articles and trials and miscalculation of caloric needs, as discussed by Janse van Mantgem et al.^70^

### Longitudinal Assessment of Energy Expenditure

Longitudinal assessments of energy expenditure were presented in 3 articles.^37^^,^^49^^,^^50^ Desport et al^49^ and Vaisman et al^50^ observed a significant decrease in mREE when measured over 6 months to 1 year. However, when mREE was expressed as a percentage of predicted REE by the HB equation, Desport et al^49^ and Bouteloup et al^37^ reported a stable metabolic state over the course of disease progression. When mREE was normalized for FFM (mREE/FFM), Vaisman et al^50^ and Bouteloup et al^37^ observed a significant increase in mREE/FFM over time,^37^^,^^50^ wherein mREE remained stable and FFM significantly declined. As we have described, FFM is the biggest determinant of REE in cross-sectional analysis.^16^^,^^17^ However, if this relationship held true over time, then a decrease in FFM should always accompany a decrease in REE. This highlights the value of longitudinal energy expenditure measurements. Further investigation is needed to better understand the longitudinal changes in energy expenditure reported in this small subset of articles; perhaps other physiological factors may have greater influence on REE with disease progression.

Further validation of predictive equations could consider longitudinal changes in body weight and composition, with a specific focus on the proportion of FFM. Holdom et al^71^ demonstrated that the stratification of the metabolic status of people living with MND is influenced by the criteria used and factors specific to the demographics of the cohort.^71^ The authors concluded that cohort-specific reference values from healthy control participants should be developed to define hyper- or hypometabolism.^71^

### Considerations

Using an organizational model such as the PRISMA-ScR, guided by the PICOS criteria, provided a robust framework to retrieve and summarize the evidence we found on the assessment of energy expenditure in people living with MND. However, there were limitations associated with conducting this scoping review. Primarily, the small body of literature captured in this review was highly influenced by 10 articles (35.7%) arising from collaborations across the same research groups (Table 1).^17^^,^^37^^,^^39^^,^^40^^,^^49^^,^^57–59^^,^^62^^,^^65^ Moreover, 3 included articles reported data from the same study, and the same study population, therefore, is presented on multiple occasions.^40^^,^^58^^,^^59^ However, because these articles used different data from this study to address different aims and objectives, the extracted data were synthesized and presented in different ways in this review. The inclusion of articles in this scoping review was restricted to those published in the English language (Figure 1). As such, 2 identified articles were excluded when the full-length articles were assessed for eligibility.^98^^,^^99^ Although this may have resulted in omission of relevant evidence in the literature, we were not able to translate articles published in other languages because of time and resource restrictions. Although it was beyond the scope of this review to conduct a full quality assessment of the included articles, we have presented the inconsistencies and missing data identified during the data extraction process.

## CONCLUSION

This review has mapped the current international approaches to assess energy expenditure in MND. IC is the most common method for estimating REE; however, there is an absence of a standardized, validated protocol for the conduction and reporting of IC protocols and outcome measurements.

Hypermetabolism is commonly identified in people living with MND by comparisons of mREE and pREE. The number of individuals classified as hypermetabolic is dependent on the predictive energy equation and the metabolic index threshold applied. This is most often the HB equation at a threshold of 10%, regardless of evidence that this equation may be inaccurate in up to 68% of an MND study population. Normalization of mREE against estimates of FFM may be more appropriate; however, this technology is not always available or practical in either a clinical or research setting. The clinical (eg, disease stage and phenotype) and anthropometric (proportion of FM and FFM) parameters of the study population also need to be considered for differences that may drive changes in the mREE and, subsequently, the metabolic index and mREE to FFM ratio. Standardization of the design and conduct and reporting of IC research would enable comparisons of REE across international databases. In turn, this would allow the stratification of individuals according to measurements of REE, opposed to the current categorization of hypermetabolism, which may be controversial.

## Supplementary Material

nuae118_Supplementary_Data
