# Supplementary material for: Mapping the Evidence for Measuring Energy Expenditure and Indicating Hypermetabolism in Motor Neuron Disease: A Scoping Review
Source: Nutr Rev. 2024 Oct 7;83(5):943–60. doi: 10.1093/nutrit/nuae118 (PMC11986331; doi:10.1093/nutrit/nuae118)
Supplement: nuae118_Supplementary_Data [file nuae118_supplementary_data.zip › nuae118_Supplementary_Data/Energy expenditure scoping review protocol 160524.docx]

**A protocol for a scoping review entitled “Mapping the evidence for measuring energy expenditure and indicating hypermetabolism in Motor Neuron Disease: a scoping review”**

**Authors**

Sarah A Roscoe PhD ^1^, Scott P Allen PhD ^1^, Christopher J McDermott PhD ^1^, Theocharis Stavroulakis PhD ^1*^

^1^ Division of Neuroscience, School of Medicine and Population Health, Sheffield Institute for Translational Neuroscience (SITraN), The University of Sheffield, Sheffield, England, S10 2HQ.

***Correspondence:** Theocharis Stavroulakis: Division of Neuroscience, School of Medicine and Population Health, Sheffield Institute for Translational Neuroscience, The University of Sheffield, 385a Glossop Road, Sheffield, England, S10 2HQ; 0114 222 2294; [t.stavroulakis@sheffield.ac.uk](mailto:t.stavroulakis@sheffield.ac.uk).

**Project Information**

Type and Method of Review

Scoping Review.

Stage of Review

Completed. Manuscript undergoing revision following reviewer comments.

Organisational Affiliation of the Review

University of Sheffield

Funding Sources/ Sponsors

This scoping review was undertaken as part of a wider study that was funded by the Department of Neuroscience at the University of Shefﬁeld, the Darby Rimmer MND Foundation and the National Institute for Health and Care Research (NIHR) Shefﬁeld Biomedical Research Centre (NIHR203321). The views expressed are those of the author(s) and not necessarily those of the NIHR or the Department of Health and Social Care. SPA is funded by the Academy of Medical Sciences (Springboard award - SBF005_1064) and the Motor Neurone Disease Association (887-791).

Conflicts of Interest

None to report

Collaborators

None to report

**Review Question and Aims**

Review Question

“What methods (i.e., devices, protocols, equations and outcome measures) have been used to measure energy expenditure (resting and total) in people living with MND?”.

Keywords

Motor neuron(e) disease, Hypermetabolism, Malnutrition, Resting Energy Expenditure, Total Daily Energy Expenditure, Indirect Calorimetry, Doubly-labelled Water, Predictive Energy Equations.

Aim

The aim of this scoping review is to map the international methods used to measure energy expenditure in adults living with MND, as well as to highlight the fundamental discrepancies when indicating hypermetabolism in the MND literature.

Objectives

The objectives were defined according to the ‘Population, Intervention, Comparator, Outcome and Study design’ (PICOS) framework (1) (Table 1).

Table 1

| **PICOS Criterion** | **Inclusion criteria** | **Exclusion criteria** |
| --- | --- | --- |
| Participants / population(s) | - Adults living with motor neuron(e) disease (≥18 years) - Studies conducted in participants with a confirmed diagnosis of MND at any stage and any MND phenotype - Studies conducted in humans | - Studies in children (<18 years) - Studies conducted in healthy participants or any condition other than MND - Non-human studies |
| Intervention(s) | - Studies that measured energy expenditure by means of indirect calorimetry, plethysmography and/or doubly labelled water. | - Studies that did not measure energy expenditure (e.g., the sole use of predictive energy equations) |
| Comparator(s) | - None/Healthy controls. | - None/Healthy controls. |
| Outcome(s) | Primary outcomes:   - Studies that describe methods, protocols and devices used to measure direct or indirect output values when measuring energy expenditure in MND (e.g., mREE, VO_2_, VCO_2,_ RQ). | - Studies that did not measure energy expenditure in MND (e.g., sole use of predictive energy equations). |
|  | Secondary outcomes:   - Studies that compare measurements of resting energy expenditure against predictions of resting energy expenditure to determine accuracy in MND; - Studies that present thresholds to indicate hypermetabolism in MND. |  |
| Study design | - Primary quantitative research journal articles; - Cross-sectional or longitudinal; - Case control or cohort; - International; - Studies available in full text; - Published in the English language; - No date restrictions; - In humans. | - Qualitative studies; - Reviews, systematic reviews, opinion pieces, editorials, letters, commentaries; - Non-English language - Studies unavailable in full text - Not in humans. |

**Methods**

This scoping review will be conducted following the five-step framework outlined by Arksey and O’Malley, i.e., 1) identification of the research question; 2) identification of primary research literature; 3) study selection; 4) data extraction; and 5) data synthesis (2).

Searches

The primary reviewer (SR) will search the following databases from inception using a combination of keywords related to the measurement of energy expenditure in motor neuron disease (MND) (Appendix 1).

- MEDLINE (Via Ovid)
- CINAHL (Via EBSCO)
- Web of Science

The search strategy, including all identified keywords and index terms, will be developed in MEDLINE and subsequently adapted for CINAHL and Web of Science. Keyword terms will be optimised using wildcards and truncations and combined with medical subject headings (MeSH) using Boolean Operators. Only studies conducted in humans and published in the English language will be included. Search results will not be limited by publication date. Reference lists of key studies will be screened by hand and ‘cited by’ articles on PubMed to identify additional studies.

Data Extraction

Study records will be extracted from the three aforementioned databases and imported into Mendeley for deduplication by the primary reviewer (SR). Results will be transformed by SR onto a Microsoft Excel spreadsheet for screening by title and abstract prior to full-text review. A hierarchical screening tool will be developed by SR and reviewed by TS to support screening (3). This will be utilised to support screening and will be visualised according to PRISMA-ScR guidance (4). All titles and abstracts will be independently assessed by a second reviewer (TS). Any discrepancies regarding title, abstract or full-text screening will be resolved via discussion with two reviewers (SR and TS). Details of excluded papers will be documented as per PRISMA-ScR guidance (4).

The following data will be extracted from eligible full-text articles by SR into Excel.

Study Identifiers:

1. Author
2. Study title
3. Year of publication
4. Location/country

Study characteristics:

1. Study design
2. Study aim(s)
3. Study centre
4. Study start/end dates

Population characteristics

1. Exclusion criteria
2. Sample size of MND participants
3. Patient-related factors (e.g., clinical characteristics, sex, age)
4. Inclusion of control participants
5. Frequency of data collection (if applicable)

Study assessments:

1. Measurement of energy expenditure
2. Assessments of nutritional status
3. Assessment of other relevant outcomes (i.e., predictions of energy expenditure, assessment of hypermetabolism, assessment of body composition).

The data extraction form will be piloted and iteratively adjusted as per data availability.

Strategy for Data Synthesis

Synthesis of quantitative data will be performed through basic descriptive analysis. Qualitative data synthesis will also be descriptive, as per the remit of a scoping review.

Risk of Bias (Quality) Assessment

An assessment of quality, critical appraisal or risk of bias will not be completed. As per the Joanna Briggs Institute methodological guidance for scoping reviews (5) this is not deemed consistent with the aims of a scoping review in mapping the available evidence.

Dissemination Plans

Publication in a peer reviewed journal.

**References**

1. Richardson S, Wilson MC, Nishikawa J, Hayward RS. The well-built clinical question: a key to evidence-based decisions. ACP J Club. 1995;123(3):A12-13.

2. Arksey H, O’Malley L. Scoping studies: Towards a methodological framework. International Journal of Social Research Methodology: Theory and Practice. 2005 Feb;8(1):19–32.

3. Polanin JR, Pigott TD, Espelage DL, Grotpeter JK. Best practice guidelines for abstract screening large-evidence systematic reviews and meta-analyses. Res Synth Methods. 2019 Sep 1;10(3):330–42.

4. Tricco AC, Lillie E, Zarin W, O’Brien KK, Colquhoun H, Levac D, et al. PRISMA extension for scoping reviews (PRISMA-ScR): Checklist and explanation. Ann Intern Med [Internet]. 2018 Oct 2 [cited 2024 May 16];169(7):467–73. Available from: https://www.acpjournals.org/doi/10.7326/M18-0850

5. Peters MDJ, Marnie C, Tricco AC, Pollock D, Munn Z, Alexander L, et al. Updated methodological guidance for the conduct of scoping reviews. JBI Evid Synth [Internet]. 2020 Oct 1 [cited 2024 May 16];18(10):2119–26. Available from: https://journals.lww.com/jbisrir/fulltext/2020/10000/updated_methodological_guidance_for_the_conduct_of.4.aspx

**Appendices**

Appendix 1: Database search strategies for Medline via Ovid, CINAHL and Web of science. Keyword terms were optimised using wildcards and truncations and combined with medical subject headings (MeSH) using Boolean Operators. Only studies conducted in humans and published in the English language were included. Search results were not limited by publication date.

| **Search** | **Terms** | **Results** |
| --- | --- | --- |
| **Medline via Ovid** | | |
| 1 | Motor neuron disease/ or Amyotrophic lateral sclerosis/ or motor neuron* disease.mp. or MND.mp. or ALS.mp. [mp=title, book title, abstract, original title, name of substance word, subject heading word, floating sub-heading word, keyword heading word, organism supplementary concept word, protocol supplementary concept word, rare disease supplementary concept word, unique identifier, synonyms] | 88,083 |
| 2 | nutritional status/ or nutrition assessment/ or nutrition therapy/ or malnutrition/ or malnutrition.mp. or nutrition* assessment.mp. or nutrition* monitoring.mp. or *nutrition/ or malnutrition.mp. [mp=title, book title, abstract, original title, name of substance word, subject heading word, floating sub-heading word, keyword heading word, organism supplementary concept word, protocol supplementary concept word, rare disease supplementary concept word, unique identifier, synonyms] | 120,403 |
| 3 | energy metabolism/ or basal metabolism/ or oxygen consumption/ or metabolism/ or *energy expenditure/ or energy demand.mp. or resting energy expenditure.mp. or REE.mp. or total daily energy expenditure.mp. or TDEE.mp. or basal energy expenditure.mp. or resting metabolic rate.mp. or RMR.mp. or basal metabolic rate.mp. or BMR.mp. or hypermetabolism.mp. or *metabolism/ [mp=title, book title, abstract, original title, name of substance word, subject heading word, floating sub-heading word, keyword heading word, organism supplementary concept word, protocol supplementary concept word, rare disease supplementary concept word, unique identifier, synonyms] | 229,297 |
| 4 | calorimetry, indirect/ or plethysmography/ or indirect calorimetry.mp. or IC.mp. or whole body air displacement plethysmography.mp. or bodpod.mp. or doubly-labelled water.mp. or DLW.mp. or predictive energy equations.mp. [mp=title, book title, abstract, original title, name of substance word, subject heading word, floating sub-heading word, keyword heading word, organism supplementary concept word, protocol supplementary concept word, rare disease supplementary concept word, unique identifier, synonyms] | 90,464 |
| 5 | 1 and 2 and 3 | 31 |
| 6 | limit 5 to (English language) | 27 |
| 7 | 1 and 3 and 4 | 29 |
| 8 | limit 7 to (English language) | 27 |
| **CINAHL** | | |
| 1 | Motor neuron disease/ or Amyotrophic lateral sclerosis/ or motor neuron* disease.mp. or MND.mp. or ALS.mp. | 7,606 |
| 2 | nutritional status/ or nutrition assessment/ or nutrition therapy/ or malnutrition/ or malnutrition.mp. or nutrition* assessment.mp. or nutrition* monitoring.mp. or *nutrition/ or malnutrition.mp. | 167,243 |
| 3 | energy metabolism/ or basal metabolism/ or oxygen consumption/ or metabolism/ or *energy expenditure/ or energy demand.mp. or resting energy expenditure.mp. or REE.mp. or total daily energy expenditure.mp. or TDEE.mp. or basal energy expenditure.mp. or resting metabolic rate.mp. or RMR.mp. or basal metabolic rate.mp. or BMR.mp. or hypermetabolism.mp. or *metabolism/ | 325,148 |
| 4 | calorimetry, indirect/ or plethysmography/ or indirect calorimetry.mp. or IC.mp. or whole body air displacement plethysmography.mp. or bodpod.mp. or doubly-labelled water.mp. or DLW.mp. or predictive energy equations.mp. | 5,761 |
| 5 | 1 and 2 and 3 and 4; limited to English language & human | 3 |
| **Web of Science** | | |
| 1 | **(((TS=("Motor neuron* disease")) OR TS=("Amyotrophic lateral sclerosis")) OR TS=("MND")) OR TS=("ALS")** | 240,119 |
| 2 | **(((((TS=("Nutrition* stat*" )) OR TS=("nutrition* assessment" )) OR TS=("nutrition* therapy")) OR TS=(malnutrition)) OR TS=("nutrition* monitoring" )) OR TS=(*nutrition)** | 3,610,276 |
| 3 | **((((((((((((((((((TS=("energy metabolism")) OR TS=("basal metabolism")) OR TS=("oxygen consumption")) OR TS=(metabolism)) OR TS=("*energy expenditure")) OR TS=("energy demand")) OR TS=("resting energy expenditure")) OR TS=("total daily energy expenditure" )) OR TS=("basal energy expenditure" ))) OR TS=(TDEE)) OR TS=(REE)) OR TS=("resting metabolic rate" )) OR TS=(RMR)) OR TS=("basal metabolic rate")) OR TS=(BMR)) OR TS=(hypermetabolism)) OR TS=(metabolism))** | 11,841,138 |
| 4 | **((((((((TS=(indirect calorimetry)) OR TS=(IC)) OR TS=(Plethysmography)) OR TS=("whole body air displacement plethysmography")) OR TS=(BODPOD)) OR TS=("Doubly labelled water")) OR TS=(DLW)) OR TS=(predictive energy equations))** | 606,872 |
| 5 | **#4 AND #3 AND #2 AND #1** | 39 |
