# Supplementary material for: Mapping the Evidence for Measuring Energy Expenditure and Indicating Hypermetabolism in Motor Neuron Disease: A Scoping Review
Source: Nutr Rev. 2024 Oct 7;83(5):943–60. doi: 10.1093/nutrit/nuae118 (PMC11986331; doi:10.1093/nutrit/nuae118)
Supplement: nuae118_Supplementary_Data [file nuae118_supplementary_data.zip › nuae118_Supplementary_Data/Roscoe et al. Supplementary Tables (1).docx]

# Supplementary Tables

Supplementary Table 1. PICOS eligibility criteria.

| **PICOS Criterion** | **Inclusion criteria** | **Exclusion criteria** |
| --- | --- | --- |
| Participants / population(s) | - Adults living with motor neuron(e) disease (≥18 years) - Studies conducted in participants with a confirmed diagnosis of MND at any stage and any MND phenotype - Studies conducted in humans | - Studies in children (<18 years) - Studies conducted in healthy participants or any condition other than MND - Non-human studies |
| Intervention(s) | - Studies that measured energy expenditure by means of indirect calorimetry, plethysmography and/or doubly labelled water. | - Studies that did not measure energy expenditure (e.g., the sole use of predictive energy equations) |
| Comparator(s) | - None/Healthy controls. | - None/Healthy controls. |
| Outcome(s) | Primary outcomes:   - Studies that describe methods, protocols and devices used to measure direct or indirect output values when measuring energy expenditure in MND (e.g., mREE, VO_2_, VCO_2,_ RQ). | - Studies that did not measure energy expenditure in MND (e.g., sole use of predictive energy equations). |
|  | Secondary outcomes:   - Studies that compare measurements of resting energy expenditure against predictions of resting energy expenditure to determine accuracy in MND; - Studies that present thresholds to indicate hypermetabolism in MND. |  |
| Study design | - Primary quantitative research journal articles; - Cross-sectional or longitudinal; - Case control or cohort; - International; - Studies available in full text; - Published in the English language; - No date restrictions; - In humans. | - Qualitative studies; - Reviews, systematic reviews, opinion pieces, editorials, letters, commentaries; - Non-English language - Studies unavailable in full text - Not in humans. |

Supplementary Table 2. Database search strategies for Medline via Ovid, CINAHL and Web of science. Keyword terms were optimised using wildcards and truncations and combined with medical subject headings (MeSH) using Boolean Operators. Only studies conducted in humans and published in the English language were included. Search results were not limited by publication date.

| **Search** | **Terms** | **Results** |
| --- | --- | --- |
| **Medline via Ovid** | | |
| 1 | Motor neuron disease/ or Amyotrophic lateral sclerosis/ or motor neuron* disease.mp. or MND.mp. or ALS.mp. [mp=title, book title, abstract, original title, name of substance word, subject heading word, floating sub-heading word, keyword heading word, organism supplementary concept word, protocol supplementary concept word, rare disease supplementary concept word, unique identifier, synonyms] | 88,083 |
| 2 | nutritional status/ or nutrition assessment/ or nutrition therapy/ or malnutrition/ or malnutrition.mp. or nutrition* assessment.mp. or nutrition* monitoring.mp. or *nutrition/ or malnutrition.mp. [mp=title, book title, abstract, original title, name of substance word, subject heading word, floating sub-heading word, keyword heading word, organism supplementary concept word, protocol supplementary concept word, rare disease supplementary concept word, unique identifier, synonyms] | 120,403 |
| 3 | energy metabolism/ or basal metabolism/ or oxygen consumption/ or metabolism/ or *energy expenditure/ or energy demand.mp. or resting energy expenditure.mp. or REE.mp. or total daily energy expenditure.mp. or TDEE.mp. or basal energy expenditure.mp. or resting metabolic rate.mp. or RMR.mp. or basal metabolic rate.mp. or BMR.mp. or hypermetabolism.mp. or *metabolism/ [mp=title, book title, abstract, original title, name of substance word, subject heading word, floating sub-heading word, keyword heading word, organism supplementary concept word, protocol supplementary concept word, rare disease supplementary concept word, unique identifier, synonyms] | 229,297 |
| 4 | calorimetry, indirect/ or plethysmography/ or indirect calorimetry.mp. or IC.mp. or whole body air displacement plethysmography.mp. or bodpod.mp. or doubly-labelled water.mp. or DLW.mp. or predictive energy equations.mp. [mp=title, book title, abstract, original title, name of substance word, subject heading word, floating sub-heading word, keyword heading word, organism supplementary concept word, protocol supplementary concept word, rare disease supplementary concept word, unique identifier, synonyms] | 90,464 |
| 5 | 1 and 2 and 3 | 31 |
| 6 | limit 5 to (English language) | 27 |
| 7 | 1 and 3 and 4 | 29 |
| 8 | limit 7 to (English language) | 27 |
| **CINAHL** | | |
| 1 | Motor neuron disease/ or Amyotrophic lateral sclerosis/ or motor neuron* disease.mp. or MND.mp. or ALS.mp. | 7,606 |
| 2 | nutritional status/ or nutrition assessment/ or nutrition therapy/ or malnutrition/ or malnutrition.mp. or nutrition* assessment.mp. or nutrition* monitoring.mp. or *nutrition/ or malnutrition.mp. | 167,243 |
| 3 | energy metabolism/ or basal metabolism/ or oxygen consumption/ or metabolism/ or *energy expenditure/ or energy demand.mp. or resting energy expenditure.mp. or REE.mp. or total daily energy expenditure.mp. or TDEE.mp. or basal energy expenditure.mp. or resting metabolic rate.mp. or RMR.mp. or basal metabolic rate.mp. or BMR.mp. or hypermetabolism.mp. or *metabolism/ | 325,148 |
| 4 | calorimetry, indirect/ or plethysmography/ or indirect calorimetry.mp. or IC.mp. or whole body air displacement plethysmography.mp. or bodpod.mp. or doubly-labelled water.mp. or DLW.mp. or predictive energy equations.mp. | 5,761 |
| 5 | 1 and 2 and 3 and 4; limited to English language & human | 3 |
| **Web of Science** | | |
| 1 | **(((TS=("Motor neuron* disease")) OR TS=("Amyotrophic lateral sclerosis")) OR TS=("MND")) OR TS=("ALS")** | 240,119 |
| 2 | **(((((TS=("Nutrition* stat*" )) OR TS=("nutrition* assessment" )) OR TS=("nutrition* therapy")) OR TS=(malnutrition)) OR TS=("nutrition* monitoring" )) OR TS=(*nutrition)** | 3,610,276 |
| 3 | **((((((((((((((((((TS=("energy metabolism")) OR TS=("basal metabolism")) OR TS=("oxygen consumption")) OR TS=(metabolism)) OR TS=("*energy expenditure")) OR TS=("energy demand")) OR TS=("resting energy expenditure")) OR TS=("total daily energy expenditure" )) OR TS=("basal energy expenditure" ))) OR TS=(TDEE)) OR TS=(REE)) OR TS=("resting metabolic rate" )) OR TS=(RMR)) OR TS=("basal metabolic rate")) OR TS=(BMR)) OR TS=(hypermetabolism)) OR TS=(metabolism))** | 11,841,138 |
| 4 | **((((((((TS=(indirect calorimetry)) OR TS=(IC)) OR TS=(Plethysmography)) OR TS=("whole body air displacement plethysmography")) OR TS=(BODPOD)) OR TS=("Doubly labelled water")) OR TS=(DLW)) OR TS=(predictive energy equations))** | 606,872 |
| 5 | **#4 AND #3 AND #2 AND #1** | 39 |
